# Phenotypic and genomic insights into alfalfa diversity: Identifying critical loci for enhanced resilience

**DOI:** 10.1002/tpg2.70155

**Published:** 2025-11-19

**Authors:** Irving Arcia‐Ruiz, Marie Pégard, Fabien Surault, Dalibor Živanov, Dragan Milić, Đura Karagić, Bernadette Julier

**Affiliations:** ^1^ INRAE, URP3F, Le Chêne Lusignan France; ^2^ Institute of Field and Vegetable Crops (IFVCNS) Novi Sad Serbia

## Abstract

Alfalfa (*Medicago sativa* L.) is a globally important forage crop, yet the relationship between its phenotypic variation and underlying genetic structure remains poorly resolved. In this study, we combined spatially adjusted, multiyear phenotyping with high‐density single‐nucleotide polymorphism genotyping in a panel of 395 cultivated accessions to (i) characterize the structure of phenotypic diversity across a broad range of agronomic traits, (ii) quantify the correspondence between phenotypic and genetic structure, and (iii) identify specific traits and genomic regions associated with genetic differentiation. Phenotypic data were collected across multiple locations and years, while genotypic data were obtained using genotyping by sequencing. Multivariate and feature selection analyses revealed nine phenotypic groups, with 78 traits confirmed as important, especially anthracnose resistance and lodging susceptibility. However, phenotypic clustering showed only moderate correspondence with genetic structure (Mantel *r* = 0.17, *p* = 0.001). Genome‐wide scans for genetic groups differentiation (*F*_ST_) identified discrete genomic regions enriched for candidate genes linked to disease resistance, stress tolerance, and reproductive processes, including loci potentially involved in self‐incompatibility. These results highlight the complexity of genotype–phenotype relationships in alfalfa and pinpoint specific genomic targets for future breeding efforts aiming to enhance resilience, productivity, and adaptability.

## Core Ideas

- American and European accessions spanned phenotypic groups, while most Chinese ones clustered in a single group.
- Genetic groups showed broad within‐group phenotypic diversity, with wide variation among accessions.
- Anthracnose resistance and lodging susceptibility are key phenotypic drivers of genetic group differentiation.
- Genome scans revealed candidate regions for disease resistance, abiotic stress, and reproductive traits.

## Plain Language Summary

Alfalfa (*Medicago sativa*) is one of the most widely grown forage crops worldwide. In this study, we examined 395 cultivated accessions across two sites and 3 years, measuring a wide range of agronomic traits such as yield, forage quality, and disease resistance. These data were combined with genome‐wide deoxyribonucleic acid markers to better understand the relationship between genetic background and observable traits. Results reveal nine phenotypic groups and 78 traits linked to genetic structure, with anthracnose resistance and lodging susceptibility playing key roles, although genetic and phenotypic clustering only partly overlapped. The study also identified genomic regions linked to disease resistance, stress tolerance, and reproduction processes. The findings provide useful targets for breeding programs aimed at developing varieties with higher productivity, stronger resistance to disease, and improved adaptation to different environments.

**Abbreviations**
ADF: acid detergent fiber
BIC: Bayesian information criterion
BWA: Burrows–Wheeler aligner
DAPC: discriminant analysis of principal components
GBS: genotyping by sequencing
GWAS: genome‐wide association study
LODG: lodging susceptibility
LRR‐RLK: leucine‐rich repeat receptor‐like kinases
MAF: minor allele frequency
MATE: multi‐antimicrobial extrusion
PCA: principal components analysis
PH: plant height
PROT: protein content
PV: plantlet vigor
Res_A: anthracnose resistance
RKE: random knot effects
SD: stand density
SE: elongation speed
SI: self‐incompatibility
SNP: single‐nucleotide polymorphism

## 1 INTRODUCTION

Sustainable agricultural production faces mounting challenges in maintaining productivity while minimizing environmental impacts. The combined pressures of climate change, biodiversity loss, and soil degradation have made the development of resilient and resource‐efficient cropping systems a global imperative (Leal Filho et al., 2023; Sher et al., 2024). Legumes play a critical role in this transition due to their ability to fix atmospheric nitrogen and produce protein‐rich biomass, particularly in temperate regions where reducing synthetic fertilizer dependence is a priority (Krause et al., 2024; Zhao et al., 2022).

Among forage legumes, alfalfa (*Medicago sativa* L.) stands out as a cornerstone species. Its perennial growth habit, high biomass yield, and nutritional quality (Atumo et al., 2021; Buxton et al., 1985; Sanderson & Wedin, 1989) have long positioned it as a strategic component of agroecological systems. By contributing to soil fertility and reducing dependence on external inputs, alfalfa aligns well with the goals of sustainable intensification (Peng et al., 2024; Singh et al., 2023).

Alfalfa's wide adaptability stems from an autotetraploid, outcrossing reproductive system, resulting in substantial genetic variation both within and among cultivars (Flajoulot et al., 2005; Herrmann et al., 2018; Julier et al., 2000; Tucak et al., 2008). This genetic complexity provides opportunities for breeding resilient, resource‐efficient cultivars but also poses unique challenges for the characterization and utilization of diversity (X. Li & Brummer, 2012).

Over the past century, breeding programs have been established in various regions of the world, each with distinct selection objectives adapted to local agronomic and economic contexts. These programs have relied on genetic material varying in degree of characterization and genetic differentiation, often reflecting historical introductions, domestication events, and local adaptation (Herrmann et al., 2018; Julier et al., 2017; Small & Jomphe, 1989). In North America, breeders have prioritized disease resistance and forage quality in a wide range of fall dormancy (Hanson et al., 1988; Lamb et al., 2006). In Europe, emphasis has been placed on persistence, forage quality, lodging resistance, and spring growth under frequent cutting regimes (Huyghe et al., 2014; Julier et al., 2017). Meanwhile, Chinese programs have focused on abiotic stress tolerance and biomass accumulation under saline, arid, and cold conditions (B. Yang et al., 2022; Z. Zhou, Li, et al., 2024). As a result, modern cultivated alfalfa consists of a mosaic of genetic pools shaped by regional breeding histories and germplasm exchanges.

Early diversity studies relied on morphological and agronomic evaluations, but these approaches often failed to capture the full extent of genetic variation, especially in a highly heterozygous, polyploid species such as alfalfa (Crochemore et al., 1998; Julier et al., 1995). The introduction of molecular markers (restriction fragment length polymorphism, simple sequence repeats, and amplified fragment length polymorphism) allowed deeper insight into genetic structure, yet genotype–phenotype associations often remained weak or inconsistent (Herrmann et al., 2018; Tucak et al., 2008). Recent advances in high‐density single‐nucleotide polymorphism (SNP) genotyping (notably genotyping by sequencing [GBS]; Elshire et al., 2011) have greatly increased the ability to resolve genome‐wide structure and enabled genome‐wide association studies (GWASs) (Annicchiarico, Nazzicari, et al., 2015; Julier et al., 2018; X. Li et al., 2014; Yu et al., 2017).

Despite these advances, the extent to which phenotypic variation, particularly for agronomic traits, is structured among cultivated accessions remains unclear. Recent genomic studies have defined groups that broadly align with geographic origin (L. Chen et al., 2021; Pégard et al., 2023), but these genetic clusters show only limited correspondence with variation in agronomic traits, raising important questions about the manifestation of genotype–phenotype relationships in alfalfa and their relevance for breeding.

This persistent inconsistency between genetic structure and phenotypic variation, compounded by the complexity of polyploid, outcrossing reproduction and historically limited investment in molecular breeding, has slowed the effective implementation of genomics‐assisted selection in alfalfa (Annicchiarico et al., 2025; X. Li & Brummer, 2012). As a consequence, genetic gains remain modest (Annicchiarico, Barrett, et al., 2015; Hawkins & Yu, 2018), and the full potential of genomic resources has yet to be realized in sustainability‐oriented breeding programs.

To address these challenges, we conducted an integrative study combining spatially adjusted, multiyear phenotyping with dense genomic data across a large and diverse panel of cultivated alfalfa. Leveraging a broad collection of accessions already described with molecular markers (Pégard et al., 2023), our objectives were to (i) characterize the phenotypic structure of cultivated alfalfa for a wide range of traits, (ii) assess the degree of correspondence between genetic and phenotypic diversity, and (iii) identify traits and genomic regions contributing most strongly to genetic group differentiation.

## 2 MATERIALS AND METHODS

### 2.1 Plant material

A total of 395 cultivated *M. sativa* L. accessions were analyzed, as originally described by Pégard et al. (2023). These comprised 311 advanced cultivars, 62 breeding or research materials, and 22 landraces, with autumn dormancy scores mostly ranging between 3 and 7. The accessions were sourced from multiple geographic regions, including Europe (314 accessions), North America (45), China (17), South America (14), the Middle East (3), and Japan (1). Detailed information on the accession origin is provided in Table S1.

### 2.2 Genetic data

Genotypic data were previously published by Pégard et al. (2023). Briefly, deoxyribonucleic acid was extracted from pooled leaflet samples collected from 100 plants per accession, and GBS was performed using *Pst*I and *Mse*I restriction enzymes, following the protocol of Julier et al. (2018), based on the method developed by Elshire et al. (2011). Reads were preprocessed using the GBprocesS bioinformatics pipeline (Schaumont, 2020) and mapped to the alfalfa reference genome (haplotype copy 2; H. Chen et al., 2020) using the Burrows–Wheeler aligner (BWA) with the BWA‐MEM algorithm. Genotype calling was conducted with a custom pipeline, and SNPs were filtered to retain only those with no more than 5% missing data, resulting in a final dataset of 227,092 markers.

Two marker subsets were derived from this dataset: the first, referred to as the 89K‐SNP‐set, contained only markers with no missing values, and the second, the 210K‐SNP‐set, included markers with up to 5% missing data (imputed based on minor allele frequency [MAF]) and excluded markers without known genomic positions (e.g., scaffolds). Both sets were filtered to remove SNPs with MAF < 0.01.

**Core Ideas**

- American and European accessions spanned phenotypic groups, while most Chinese ones clustered in a single group.
- Genetic groups showed broad within‐group phenotypic diversity, with wide variation among accessions.
- Anthracnose resistance and lodging susceptibility are key phenotypic drivers of genetic group differentiation.
- Genome scans revealed candidate regions for disease resistance, abiotic stress, and reproductive traits.

### 2.3 Phenotypic data

Phenotypic data were obtained from multiyear field trials conducted at two locations: Lusignan, France (46°23′60″ N, 0°4′48″ E), and Novi Sad, Serbia (45°15′0″ N, 19°51′0″ E), as detailed by Pégard et al. (2023). Trials were established in spring 2018 using an augmented block design with four incomplete blocks arranged in 44 columns and 10 rows, totaling 440 plots. Initially, 400 accessions were included, but after storm damage in Lusignan and seed shortages, 387 accessions were resown in a second trial established in August 2018. Replication number varied among accessions: five were replicated six times, 15 were evaluated twice in Lusignan‐May and Novi Sad, and 28 were evaluated twice in Lusignan‐August. Data were collected over multiple years, including 2018–2020, with an additional evaluation year in Lusignan in 2021.

Phenotypic evaluations were conducted on several traits, including flowering date, plant height (PH), dry matter yield, lodging susceptibility (LODG), biochemical composition related to feeding value, seed yield components, and autumn dormancy. The flowering date was recorded in the first trial sown in Lusignan during spring 2018 and expressed as the sum of degree‐days calculated from the sowing date to the flowering date, using daily mean temperatures. Plantlet vigor (PV) and density were scored 1 month after establishment in the Lusignan‐August and Novi Sad trials. In both trials, PH was measured two to five times during each regrowth period in 2019–2021, with the last measurement taken just before each cut.

Three height measurements per plot were obtained using electronic rulers for plants below 35 cm and conventional rulers otherwise, selecting individuals randomly in dense stands and focusing on the most developed plants in degraded plots. PH was also measured using image data acquired from drone flights (Surault et al., 2021). Dry matter yield was recorded at each cut in each year. Autumn dormancy was visually assessed in Lusignan in 2019, using a 1–11 scale based on regrowth height; evaluation was not possible in 2020 due to a dry autumn.

For each of the first two cuts in 2019–2021, a dry matter sample was collected from each plot, dried, ground to pass through a 1‐mm sieve, and analyzed via near‐infrared spectroscopy to predict protein and acid detergent fiber (ADF) contents (*R* ^2^ = 97.2 and 97.9, standard error of cross‐validation = 0.973 and 0.673, respectively). LODG was scored when lodging was observed, on a scale from 1 (*all the plants flattened*) to 9 (*no lodging*). Growth habit was scored in Lusignan in 2020 on a 1–9 scale, from prostrate to erect. Stand density (SD) was scored in Lusignan in 2020 and 2021 on a 1–9 scale, from no emerged plants to a full stand. No specific disease outbreaks were observed during the trials.

Seed yield components were measured in summer 2018 on the trials in Lusignan‐spring and Novi Sad. Thirty large inflorescences at the seed maturity stage (Bolaños‐Aguilar et al., 2000) were sampled per plot, and the total number of pods, number of seeds, total pod weight, and total seed weight were measured.

Resistance to anthracnose caused by *Colletotrichum trifolii* was assessed under controlled conditions using the French strain C86‐2. After plant inoculation, the percentage of resistant plants was determined following the protocol described in Pégard et al. (2025).

Several derived variables were calculated from raw measurements. Stem elongation speed (SE) was estimated in Lusignan as the slope of the linear regression of PH against accumulated degree‐days above 0°C. Combined yield across seasons, years, and locations was estimated using a mixed model that accounted for location and year effects. For protein and ADF contents, a combined value was calculated by averaging the protein and ADF contents of spring and summer cuts when possible. For seed yield components, the number of pods per inflorescence, number of seeds per pod, number of seeds per inflorescence, thousand seed weight, pod weight per inflorescence, and seed weight per inflorescence were calculated. The full list of traits and measurements is provided in Table S2.

### 2.4 Phenotypic adjustment

All trait measurements from the trials were independently adjusted for field microenvironmental heterogeneity using the breedR package (Muñoz & Sanchez, 2020). To account for spatial heterogeneity at the plot level within trials, a random effect was modeled using the tensor product of two B‐splines bases with a covariance structure for the random knot effects (RKE). This approach addressed spatial variability along the rows and columns of the field design (Cantet et al., 2005; Cappa & Cantet, 2007; Cappa et al., 2015; Robbins et al., 2012). For each year and location, a genomic‐based mixed model was employed. The genomic estimated breeding values for each trait were calculated using the best genomic best linear unbiased prediction model (Meuwissen et al., 2001; Whittaker et al., 2000):

y=μ+Zu+Ws+ε

where y represents the raw phenotypes; μ the global mean; u the vector of random additive effects following $N(0,G\sigma_{a}^{2})$, with $\sigma_{a}^{2}$ being the additive variance and G the genomic relationship matrix between accessions; *s* the vector of random spatial effects containing the parameters of the B‐splines tensor product following $N(0,S\sigma_{s}^{2})$, with $\sigma_{s}^{2}$ being the variance of the RKE for rows and columns and S the covariance structure in two dimensions; ε is the vector of residual effects following $N(0,I\sigma_{e}^{2})$, with $\sigma_{e}^{2}$ being the residual variance. The design matrices Z and W are identity matrices linking the plots to the random effects. The method for obtaining the genomic relationship matrix G is detailed in the following section. B‐splines were anchored at a specific number of knots for rows and columns, with a higher number of knots smoothing the surfaces. breedR optimized the knot numbers through an automated grid search based on the Akaike information criterion (Akaike, 1974). The microenvironmental plot effect was removed from the observed phenotype to yield a spatially adjusted phenotype. For repeated accessions, an accession mean of the spatially adjusted phenotypes was calculated for each trait.

### 2.5 Relationship Matrix Estimation

The genomic relationship matrix (**G**) was based on VanRaden (2008), adapted to use allele frequencies instead of allele dosage (Ashraf et al., 2014). The genotyping matrix (**M**) was normalized by the minimum allele frequency (*P*) to obtain the normalized genotyping matrix (**Z**), which was then used to compute G as follows:

$$G=\frac{ZZ^{\prime }}{\frac{1}{n}\sum_{j=1}^{m}p_{j}\left(1-p_{j}\right)}$$

The denominator serves as a scaling parameter, representing the sum of the expected SNP variance across genotypes (Ashraf et al., 2014). Here, m denotes the number of markers, $p_{j}$ the frequency of the jth marker, and n a scaling number to achieve a diagonal close to 1. This approach has been recommended in previous studies on polyploid species (Ashraf et al., 2014; Cericola et al., 2018), where n=16 resulted in a diagonal mean close to 1.

Following phenotypic adjustment, all phenotypic variables were standardized through centering and scaling using the scale() function in R (R Core Team, 2024) prior to all analyses.

### 2.6 Phenotypic structure

To characterize the structure of phenotypic variation, we performed a discriminant analysis of principal components (DAPC) (Jombart et al., 2010) on the spatially adjusted phenotypic dataset, without any a priori groupings, using the adegenet package in R (Jombart, 2008; Jombart & Ahmed, 2011). DAPC allowed us to identify phenotypic clusters and to determine the traits contributing to group separation.

Phenotypic groups were defined via *k*‐means clustering, which assigns individuals to *k* clusters by maximizing between‐group variance. The optimal number of clusters was selected based on the lowest Bayesian information criterion (BIC). Traits contributing most strongly to phenotypic differentiation were identified based on their loadings along the discriminant axes. The distribution of accessions was visualized in a two‐dimensional space defined by the first two principal components using the ggplot2 package (Wickham, 2016).

### 2.7 Relationship between phenotypic and genetic diversity

To assess the correspondence between genetic structure and phenotypic diversity, we projected the genetic clusters defined by Pégard et al. (2023) onto the phenotypic principal components analysis (PCA) plot. This provided a visual framework for evaluating how genetic groupings align with patterns of phenotypic diversity.

In addition, we performed a Mantel test (Mantel, 1967; Smouse et al., 1986) using Spearman's rank correlation to quantify the association between genetic and phenotypic distance matrices. Statistical significance was evaluated by generating a distribution of correlation values under the null hypothesis of no association, through permutations of the rows and columns of one matrix. The test was implemented using the vegan package in R (Oksanen et al., 2024), with 999 permutations.

Two symmetrical n×n distance matrices were constructed, where n is the number of accessions. These matrices contain the pairwise distances between all accessions, computed separately for genotypic and phenotypic data as follows:

The genetic distance matrix was calculated using the 89K‐SNP‐set, applying Euclidean distance to the allele frequency profiles. The distance between two accessions was defined as follows:

$$d\;\left(x,y\right)=\sqrt{\sum_{i=1}^{m}{\left(x_{i}-y_{i}\right)}^{2}}$$

where $x_{i}$ and $y_{i}$ are the allele frequencies at marker i in accessions x and y, respectively, and m is the total number of markers.

The phenotypic distance matrix was computed using the Mahalanobis distance (Mahalanobis, 1936), which accounts for the covariance structure among the 217 phenotypic variables. This distance is defined as follows:

$$d\;\left(Z_{i},Z_{j}\right)=\sqrt{{\left(Z_{i}-Z_{j}\right)}^{T}S^{-1}\left(Z_{i}-Z_{j}\right)}$$

where $Z_{i}$ and $Z_{j}$ are the vectors of phenotypic variables for accessions i and j, and $S^{-1}$ is the inverse covariance matrix of the phenotypic variables.

### 2.8 Phenotypic traits influencing genetic group differentiation

To identify phenotypic traits associated with genetic group differentiation, we employed the Boruta feature selection method, a wrapper algorithm based on the random forest classifier, implemented in the R package Boruta (Kursa & Rudnicki, 2010). This approach compares the importance of observed variables to that of permuted “shadow” variables, offering a robust framework for identifying relevant predictors.

Boruta was applied to phenotypic data to predict genetic group membership, with a significance threshold of p<0.05 and up to 1000 iterations. To control for multiple testing, a Bonferroni correction was applied (Bonferroni, 1936). To minimize redundancy among predictors, highly correlated traits (Pearson's |r| > 0.9) were removed prior to running Boruta. Variables were classified as confirmed, tentative, or rejected based on their importance relative to shadow features.

### 2.9 Pairwise genomic differentiation between genetic groups

To quantify genetic differentiation between groups, we computed genome‐wide pairwise $F_{\mathrm{ST}}$ for each SNP using the 210K‐SNP‐dataset. $F_{\mathrm{ST}}$ values were computed following the methods of Nei (1973), based on allele frequencies in each group.

For each of the 15 possible pairwise comparisons among the six main genetic groups (excluding G1_Falcata, which comprised only two accessions), we calculated total heterozygosity $\left(H_{T}\right)$ and within‐group heterozygosity $\left(H_{S}\right)$ as follows:

$$H_{T}=2\bar{p}\left(1-\bar{p}\right)$$

$$H_{S}=\frac{1}{2}\left[2p_{1}\left(1-p_{1}\right)+2p_{2}\left(1-p_{2}\right)\right]$$

where $p_{1}$ and $p_{2}$ are the allele frequencies in each group, and $\bar{p}=\frac{p_{1}+p_{2}}{2}$ is the average allele frequency. The fixation index was then calculated as follows:

$$F_{\mathrm{ST}}=\frac{H_{T}-H_{S}}{H_{T}}$$

To identify genomic regions exhibiting exceptional levels of genetic differentiation, we implemented a custom, nonparametric approach based on ranking $F_{\mathrm{ST}}$ values. A single set of putatively neutral SNP was sampled and used to build an empirical null distribution of $F_{\mathrm{ST}}$ for every comparison. We then calculated the empirical probability (*p*‐value) of each genome‐wide SNP exceeding this distribution, followed by false discovery rate correction for multiple testing (Benjamini & Hochberg, 1995). SNPs with *q*‐values < 0.05 were classified as outliers, indicating loci with significantly elevated differentiation beyond neutral expectations. These loci were considered candidates for divergent selection or potential anthropogenic influence. Similar approaches have been proven to be a robust alternative to parametric outlier detection methods (Lotterhos & Whitlock, 2015).

To identify putative candidate genes, we intersected the genomic coordinates of outlier SNP with gene annotations from the XinJiangDaYe genome assembly (H. Chen et al., 2020) using the myGenomeBrowser platform (Carrere & Gouzy, 2017). We considered genes containing the outlier SNP or located within a 500 bp window upstream or downstream of the SNP. Functional annotations for candidate genes were retrieved from the integrated InterPro database in myGenomeBrowser, allowing us to assign putative functions based on conserved protein domains.

## 3 RESULTS

### 3.1 Phenotypic structure revealed by DAPC

DAPC, performed on 217 phenotypic variables, revealed a complex underlying structure among accessions. The analysis identified nine phenotypic clusters, with the optimal number of groups (*k* = 9) determined by the lowest BIC (BIC = 1985.7; Figure S1 in Supporting Information File S1).

**FIGURE 1:**
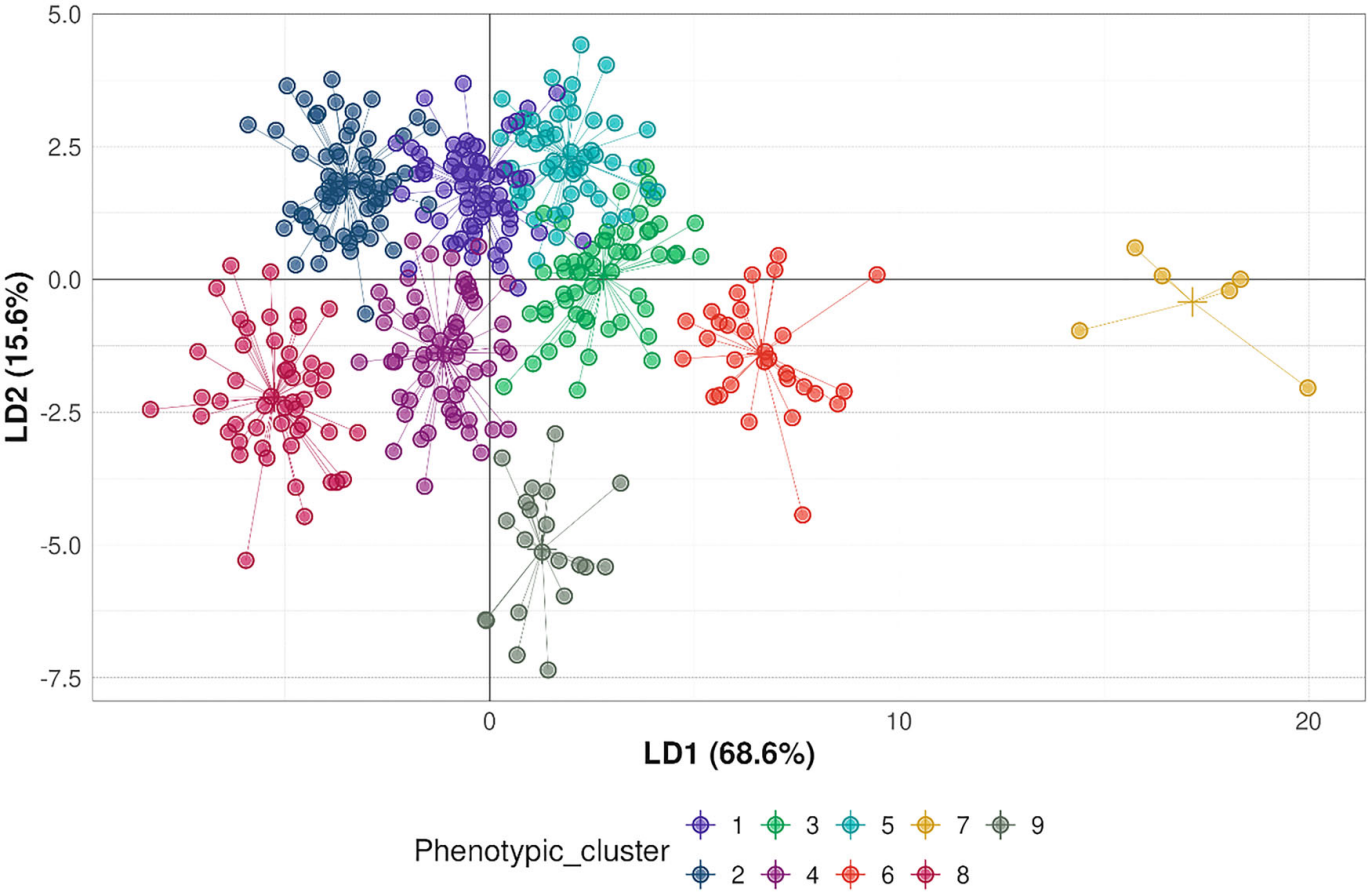
Projection of the 395 accessions onto the first two discriminant axes from the discriminant analysis of principal components (DAPC) based on phenotypic data. The two axes explain 84.1% of the total phenotypic variance. Accessions are color‐coded by their phenotypic groups as identified by *k*‐means clustering.

The first two discriminant axes explained 84.2% of the total phenotypic variance (68.6% and 15.6% for Axes 1 and 2, respectively), indicating strong structure but unequal group separation. Notably, Groups 6–9 showed well‐defined clustering, while Groups 1–5 exhibited substantial overlap (Figure 1), suggesting a gradient of phenotypic variation rather than fully discrete classes. The proportion of variance explained by the first 10 axes is shown in Figure S2 in Supporting Information File S1.

Traits most strongly associated with differentiation along Axis 1 included PH (PHd_18.05.20.L [where PHd stands for plant height measured with drone]), LODG (LODG_30.06.21.L), PV (PV_04.03.21.L), and SD (SD_24.06.20.L). For Axis 2, key discriminating traits were anthracnose resistance (Res_A), LODG (LODG_17.07.19.N), PV (PV_26.09.18.L), and PH (PH4_17.07.19.N) (Supporting Information File S1, Figure S3). Axis 3, which explained an additional 10.1% of the variance, was mainly driven by stem SE (SE_28.07.21.L) and PH (PH4_02.06.20.N), though phenotypic group separation along this axis was less distinct, except for Group 7, which remained clearly separated (Supporting Information File S1, Figure S4).

Notably, traits related to forage quality (e.g., protein content [PROT] and ADF) contributed little to group differentiation along the major axes, indicating that phenotypic structure is predominantly shaped by growth‐related and disease resistance traits.

### 3.2 Correspondence between genetic and phenotypic clusters

**FIGURE 2:**
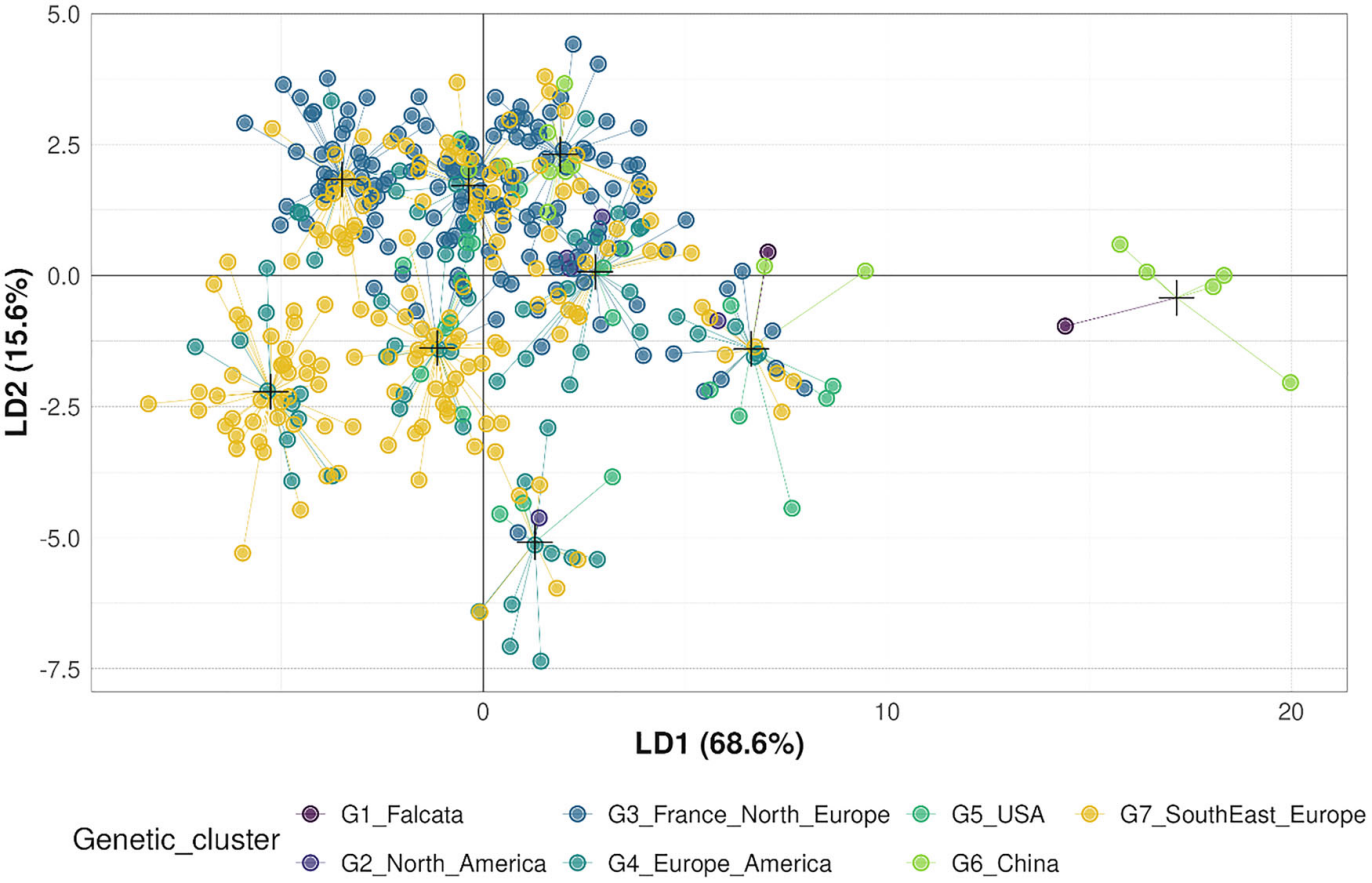
Projection of the 395 accessions onto the first two discriminant axes from the discriminant analysis of principal components (DAPC) based on phenotypic data. Accessions are color‐coded according to the seven genetic clusters defined by Pégard et al. (2023), highlighting the correspondence between genetic and phenotypic diversity.

To explore the relationship between genetic and phenotypic structures, the genetic groups defined by Pégard et al. (2023) were projected onto the phenotypic space defined by the first two axes of PCA (Figure 2).

While some correspondence was observed, overall alignment was partial. Specifically, the G6_China genetic group aligned predominantly with Phenotypic Group 2, suggesting strong genotypic–phenotypic congruence. In contrast, genetic groups G1_Falcata and G5_USA showed broader distributions across multiple phenotypic groups, indicating greater phenotypic heterogeneity within these genetic clusters. Other genetic groups also exhibited substantial overlap across phenotypic groups, reinforcing the presence of high within‐group phenotypic diversity.

These visual observations were supported by a Mantel test, which showed a significant but moderate correlation between genetic and phenotypic distance matrices (Mantel statistic = 0.168, *p* = 0.001). As illustrated in Figure S5 (Supporting Information File S1), the relationship, although statistically significant, was characterized by considerable scattering, indicating that the genetic–phenotypic association is present but not particularly strong nor consistent across the dataset.

### 3.3 Phenotypic traits influencing genetic group differentiation

Highly correlated traits (Pearson's |*r*| > 0.9) were removed prior to running Boruta, so 173 traits over 217 were kept. The Boruta feature selection analysis identified a considerable number of phenotypic traits associated with genetic group differentiation: 78 traits were classified as Confirmed, 8 as Tentative, and 87 as Rejected with respect to their contribution to group structure.

The six traits with the highest importance scores were resistance to anthracnose (Res_A), two indicators of LODG (LODG_17.07.19.N and LODG_12.06.19.N), one measure of PH (PH3_26.06.19.N), and two indicators of PROT (PROT_28.04.20.N and PROT_20.N). The full list of traits, along with their mean importance scores and Boruta classifications, is provided in Table S3.

**FIGURE 3:**
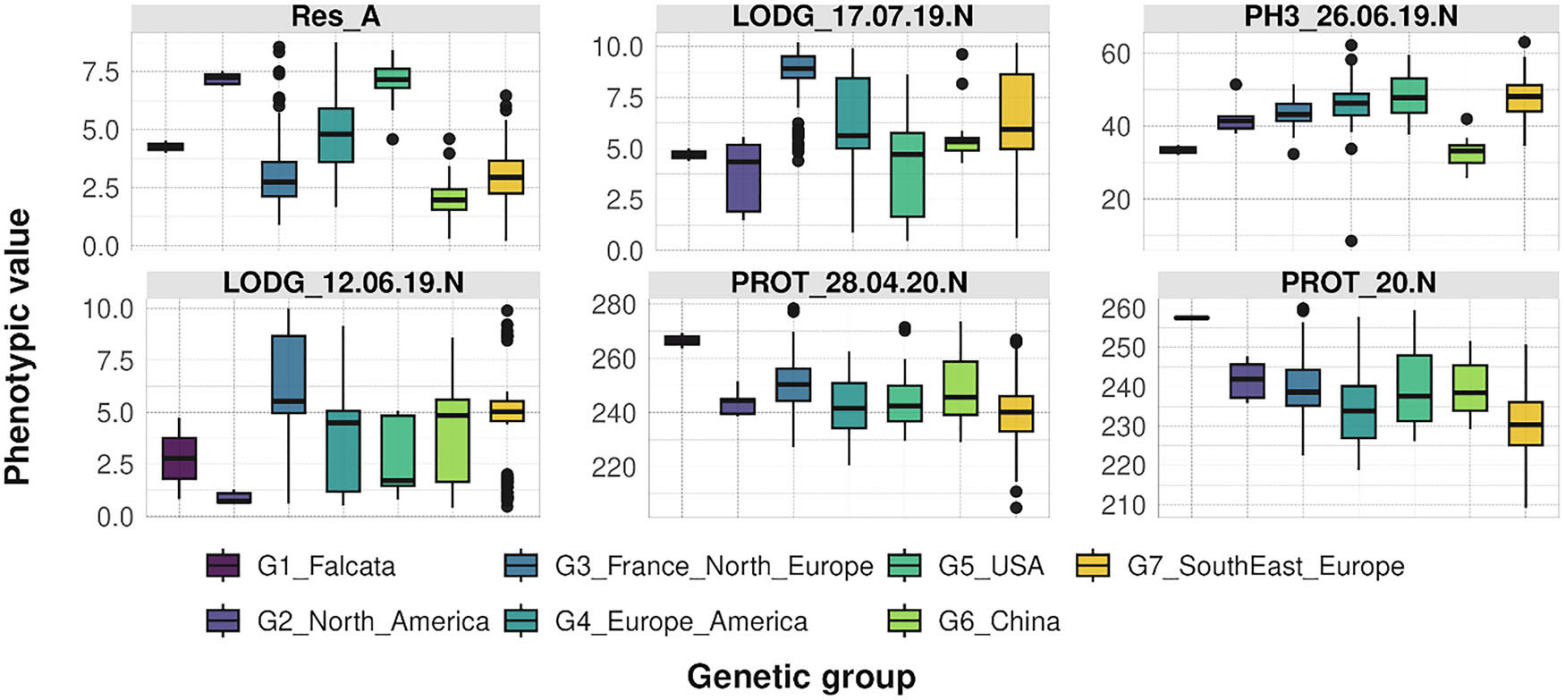
Boxplots of phenotypic values per genetic group for the six most influential traits in genetic differentiation. Variation in resistance to anthracnose (Res_A), lodging sensitivity (LODG_17.07.19.N and LODG_12.06.19.N), plant height (PH3_26.6.19.N), and protein content (PROT_28.4.20.N and PROT_20.N) across genetic groups. The numbers represent specific measurement dates, and the letters “N” and “L” indicate the locations: “N” for Novi Sad (Serbia) and “L” for Lusignan (France).

Variation in these six traits across genetic groups is shown in Figure 3. Notably, accessions from the G5_USA and G2_North_America groups exhibited the highest levels of Res_A but were also among the most susceptible to lodging (LODG_17.07.19.N and LODG_12.06.19.N). In contrast, the G3_France_North_Europe group showed the greatest lodging resistance. Differences in PH and PROT among genetic groups were less pronounced. However, G1_Falcata, despite comprising only two accessions, displayed distinctly high PROT but low PH.

Overall, these results indicate that disease resistance and LODG are the traits most strongly associated with genetic group differentiation in this panel, while variation in quality‐related traits is less pronounced among groups.

### 3.4 Pairwise genomic differentiation between genetic groups and candidate genes in highly differentiated regions

Genome‐wide pairwise $F_{\mathrm{ST}}$ analysis across 15 pairwise group comparisons revealed a number of SNPs with significantly elevated differentiation, indicative of genomic regions potentially under divergent selection or anthropogenic influence (Table S4). These outlier SNPs were distributed across all eight chromosomes but were particularly enriched on chromosomes 5 and 8, where some loci were identified as outliers in multiple group comparisons.

**FIGURE 4:**
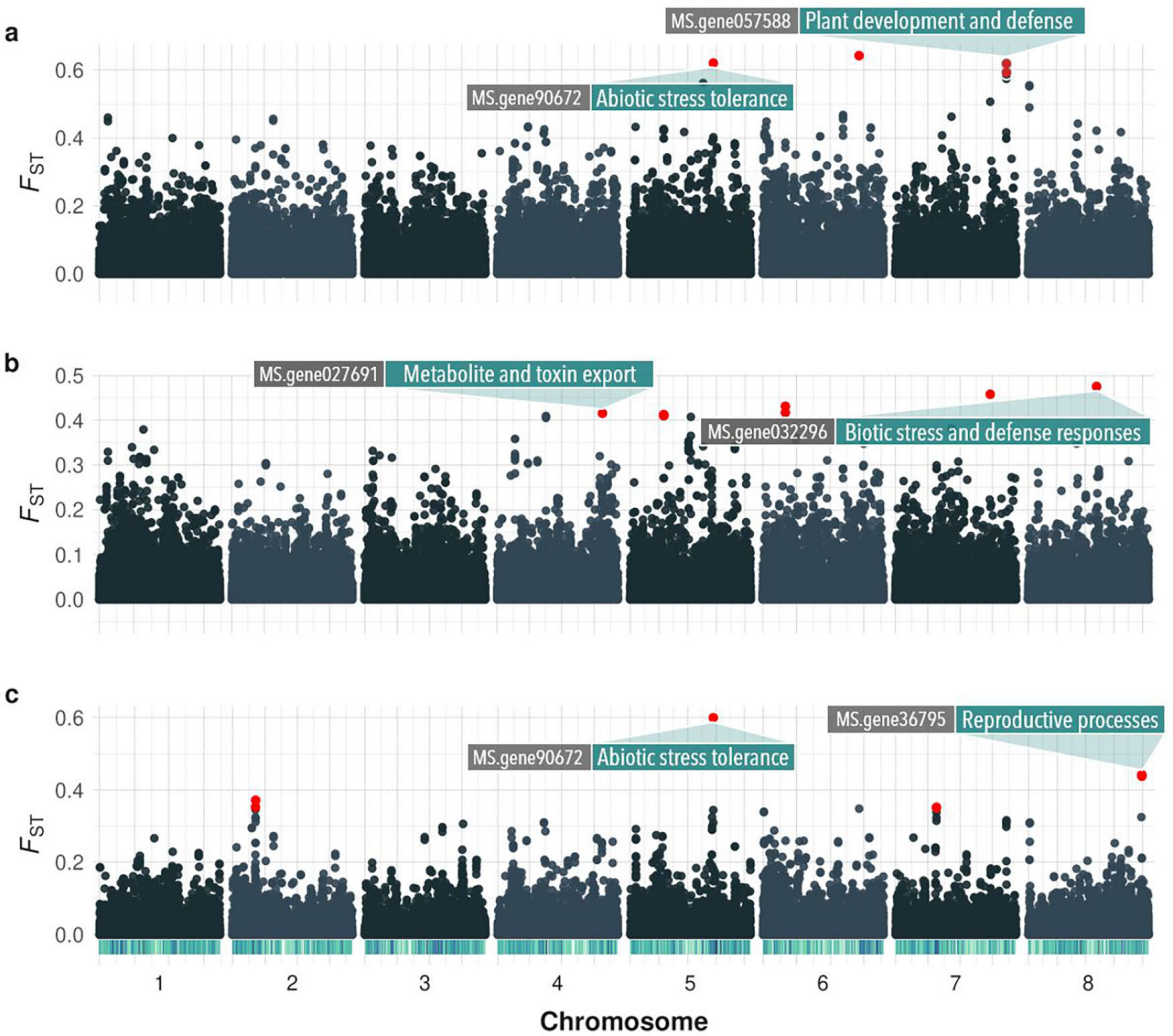
Genomic differentiation between genetic groups based on *F*_ST_ outliers. (a) G2_North_America versus G6_China, (b) G2_North_America versus G3_France_North_Europe, and (c) G3_France_North_Europe versus G6_China. Each point represents the *F*_ST_ value at a specific single‐nucleotide polymorphism (SNP). Red points indicate SNPs identified as outliers (*q*‐value < 0.05) based on an empirical null distribution and false discovery rate (FDR) correction. Labeled SNPs are located within annotated genes in the *Medicago sativa* genome and are associated with putative candidate genes.

Several of the most highly differentiated genomic regions contained annotated genes related to plant growth, defense, abiotic stress tolerance, and reproduction. For example, outlier loci were associated with genes involved in plant development and defense responses (*MS.gene027691*, *MS.gene057588*, *MS.gene032296*, and *MS.gene062909*), abiotic stress adaptation (*MS.gene90672* and *MS.gene032296*), and reproductive processes (*MS.gene36795*) (Figure 4, Table S4).

Patterns of genomic differentiation were most pronounced in comparisons involving North American, European, and Chinese groups, where distinct clusters of outlier SNP highlighted regions enriched in candidate genes of agronomic relevance (Figure 4). For example, regions differentiating North American and Chinese accessions were enriched for defense and stress‐related genes, whereas regions differentiating North American and European accessions were associated with biotic stress and defense response. Finally, European–Chinese comparisons identified outliers linked to abiotic tolerance and reproductive processes. The number and distribution of outlier SNP per comparison, as well as their associated gene annotations, are summarized in Table S4.

## 4 DISCUSSION

In this study, we investigated the relationship between phenotypic diversity and genetic structure in cultivated alfalfa by integrating multiyear spatially adjusted phenotypic data with high‐density SNP data. By analyzing the same set of accessions at both levels, we were able to directly compare patterns of phenotypic and genetic structure, and to identify specific traits and genomic regions associated with genetic group differentiation. This integrative approach offers a more comprehensive perspective on genotype–phenotype relationships in alfalfa than previous studies (Crochemore et al., 1998; Herrmann et al., 2018; Julier et al., 1995; Prosperi et al., 2006; Tucak et al., 2008), which were often limited by the number of markers, traits, or sampled individuals.

### 4.1 Phenotypic and genetic structure: Patterns and relationships

The phenotypic analysis of 395 cultivated alfalfa accessions revealed complex patterns of diversity and partially structured groups, as shown by DAPC, which identified nine clusters with substantial overlap and no clear correspondence to geographic origin.

Growth‐related traits, such as PH, PV, and SD, dominated the primary axes of differentiation, consistent with previous studies using smaller panels or fewer markers (Crochemore et al., 1998; Herrmann et al., 2018; Julier et al., 1995; Prosperi et al., 2006; Tucak et al., 2008). However, our study also underscores a substantial contribution of functional traits, particularly disease resistance (notably to *C. trifolii*) and LODG, which were less emphasized or not explicitly addressed in earlier analyses. The observed overlap among groups aligns with the decentralized nature of alfalfa breeding programs, where similar selection goals or convergent environmental pressures in distinct breeding pools may lead to analogous expression patterns for agronomic traits across diverse genetic backgrounds. This pattern is further consistent with the long history of recombination and introgression among alfalfa gene pools, as well as its biology as an outcrossing autotetraploid (Julier et al., 1995; Prosperi et al., 2006).

Despite significant clustering observed in both phenotypic (DAPC) and genotypic spaces (Pégard et al., 2023), their correspondence was weak, confirming previous results obtained with fewer markers and traits (Crochemore et al., 1998; Herrmann et al., 2018; Tucak et al., 2008). This limited correspondence likely reflects the polygenic and sometimes epistatic nature of most agronomic traits evaluated in this study, meaning that multiple genetic combinations can yield similar phenotypes, as well as the high genetic diversity maintained by the predominantly outcrossing reproductive system of cultivated alfalfa.

While accessions of one genetic group, G6_China, exhibited relatively consistent phenotypic profiles, possibly due to shared breeding practices and regionally targeted selection objectives (B. Yang et al., 2022), most of the groups displayed a broader range of phenotypic variation, suggesting more diverse selection histories or potential sampling effects in our study.

In addition, we acknowledge that our dataset is skewed toward European accessions. This sampling bias may limit the global extrapolation of our findings. Future work with more balanced representation across regions will be essential to fully capture worldwide diversity in alfalfa.

The Mantel test further supports this moderate association between genetic and phenotypic variation, in line with earlier reports in alfalfa (Herrmann et al., 2018) and other forage species, such as ryegrass (Roldán‐Ruiz et al., 2001). Notably, even in well‐studied diploid crops, as maize, strong alignment between genetic and phenotypic distances is not always observed (Flint‐Garcia et al., 2005), underscoring the inherent complexity of genotype–phenotype relationships in outcrossing species.

### 4.2 Phenotypic traits influencing genetic group differentiation

Although the overall correspondence between genotype and phenotype was limited, our combined use of spatially adjusted, multiyear phenotyping and high‐density SNP genotyping enabled a detailed dissection of trait contributions to genetic group differentiation. Notably, by applying both multivariate analyses (PCA/DAPC) and robust feature selection methods (Boruta), we identified resistance to anthracnose, LODG, PH, and PROT as the primary traits distinguishing genetic groups, traits of clear agronomic relevance in alfalfa (Annicchiarico, Barrett, et al., 2015).

Res_A, in particular, showed strong differentiation between groups, with American groups exhibiting the highest resistance levels. This aligns with American breeding programs, where resistance to *C. trifolii* has long been a priority due to its impact on plant survival and subsequent negative effect on biomass yield and forage quality (Elgin et al., 1981; Lenssen et al., 1991), and where a broader diversity of *C. trifolii* has necessitated the development of cultivars with resistance to multiple forms of the pathogen, while in Europe, resistance breeding efforts generally target the main local strains of the pathogen (Pégard et al., 2025). In contrast, Chinese breeding programs have historically focused on abiotic stress resistance, such as cold, drought, and salinity tolerance, while disease resistance has received comparatively less attention (Shi et al., 2010; B. Yang et al., 2022), a trend further compounded by the relatively recent emergence of anthracnose in China (W. Zhou, Lan, et al., 2024).

Interestingly, American groups showed high susceptibility to lodging. This pattern may also result from differences in breeding priorities, as lodging resistance has not been a primary target in America where harvest dates in spring are earlier than in Europe. In contrast, accessions from Europe showed the highest lodging resistance, likely reflecting breeding objectives under infrequent cutting regimes, where upright growth and standability are essential for efficient harvesting and for maximizing both biomass and protein production (Huyghe et al., 2014). Notably, the Flamand (or Flemish) types, well known in northern Europe for their robust, thick stems, may contribute to this increased lodging resistance, as stem thickness is a key trait associated with upright growth and mechanical stability in alfalfa.

PH and PROT also contributed to group differentiation, albeit with less contrast across groups. The high PROT observed in G1_Falcata is consistent with previous reports on *M. sativa* ssp. *falcata*, which is known for its forage quality and adaptation to marginal environments (Prosperi et al., 2006). However, as noted by previous studies, this protein advantage may be confounded by slow growth and limited biomass accumulation of this material (X. Li & Brummer, 2009). Further physiological and yield‐based evaluation would be necessary to validate the breeding potential of these genotypes.

Overall, these results highlight how differences in regional priorities and breeding histories have shaped the distribution of traits in alfalfa germplasm worldwide.

### 4.3 Genomic differentiation and candidate genes

Beyond the broad patterns of genetic structure, a genome‐wide scan for regions of elevated genetic differentiation $F_{\mathrm{ST}}$ among cultivated alfalfa revealed several candidate genomic regions putatively shaped by divergent selection or local adaptation. Notably, outlier loci overlapped with annotated genes spanning a range of agronomically relevant functions, including defense, stress adaptation, detoxification, and reproductive compatibility.

Among the most prominent gene families identified within outlier regions were leucine‐rich repeat receptor‐like kinases (LRR‐RLK), ABC transporters, glyoxalases, multi‐antimicrobial extrusion (MATE) proteins, chorismate‐utilizing enzymes, and S‐locus glycoprotein domains. The central roles of these families in plant adaptation, immunity, and development have been well established in *Medicago* and other crops (Banasiak et al., 2013; De Lorenzo et al., 2009; Ghosh, 2017; Liu et al., 2022; Pastacaldi et al., 2024; R. Yang et al., 2024). For instance, LRR‐RLK have been implicated in sensing both biotic and abiotic signals and mediating root adaptation to salinity in *M. truncatula* (De Lorenzo et al., 2009).

ABC transporters, especially those in the G subfamily, are known to modulate isoflavonoid levels and play key roles in pathogen resistance, metabolite transport, and drought response in *Medicago* (Banasiak et al., 2013; R. Yang et al., 2024). In particular, *MsABCG1* in *M. sativa* has recently been shown to confer enhanced drought tolerance when overexpressed in tobacco, underscoring its adaptive significance (R. Yang et al., 2024), and *MtABCG10* has been shown to regulate isoflavonoid accumulation and modulate pathogen resistance in *M. truncatula* (Banasiak et al., 2013).

Glyoxalases, especially Glyoxalase I and II, are upregulated under drought and salinity, and contribute to the detoxification of methylglyoxal, a cytotoxic byproduct that accumulates under stress (Ghosh, 2017). They have also been linked to reproductive functions such as pollen development and pollination responses in plants (Sankaranarayanan et al., 2017). While MATE transporters (MATE proteins) and chorismate‐utilizing enzymes contribute to the biosynthesis and extrusion of antimicrobial compounds and secondary metabolites important for defense and plant–microbe interactions (Kranawetter et al., 2021; Pastacaldi et al., 2024; Tzin & Galili, 2010).

Interestingly, some outlier regions harbored S‐locus glycoprotein domain genes implicated in pollen recognition and compatibility. *M. sativa* is a predominantly outcrossing species, primarily due to strong inbreeding depression and floral morphology that favors cross‐pollination. However, the molecular basis of pollen–pistil interactions in this species remains incompletely resolved.

Classical studies reported partial self‐incompatibility (SI), with abnormal pollen tube development and reduced seed set in self‐ or intragroup crosses (Bauchan et al., 1990; Brink & Cooper, 1938; Campbell & He, 1997). Recent transcriptomic analyses have identified several candidate genes associated with SI in *M. sativa* (L. Li et al., 2024). However, SI in alfalfa is still poorly understood, although the canonical S‐locus system, as characterized in Brassicaceae, has not been definitively confirmed. Thus, in our study, the detection of $F_{\mathrm{ST}}$ outliers in these regions may reflect ongoing divergence in compatibility factors, potentially playing a key role in pre‐breeding, hybridization, and gene flow between distinct germplasm pools. Notably, SI systems, while presenting certain challenges, also offer unique opportunities to promote cross‐pollination and harness hybrid vigor (heterosis) by minimizing self‐fertilization and inbreeding depression.

While certain highly differentiated loci may align with phenotypic traits evaluated in our study, most notably Res_A, the recently identified quantitative trait loci for Res_A detected via GWAS using the same dataset (Pégard et al., 2025) did not overlap with the loci showing the highest differentiation in our $F_{\mathrm{ST}}$ analysis. This discrepancy between GWAS‐derived loci and high‐$F_{\mathrm{ST}}$ regions is neither unusual nor problematic. Rather, it underscores the complementary nature of these approaches: GWAS directly identifies loci associated with trait variation while statistically controlling for population structure, whereas $F_{\mathrm{ST}}$ analyses highlight genomic regions contributing to divergence between distant genetic groups (Afzal et al., 2019; Labastida et al., 2023). Together, these analytical strategies offer distinct yet synergistic insights, collectively enhancing the identification of robust candidate regions for breeding and further research.

Although our dataset is robust, the lack of phenotypic assays across a broader range of abiotic conditions, pathogens, and especially for reproductive compatibility currently limits our ability to assign functional roles to candidate genes identified in outlier regions. To directly validate the effects of these differentiated loci, future work should include not only targeted phenotyping under controlled biotic and abiotic stress conditions but also the creation or selection of panels of genotypes carrying different alleles or dosages at the focal SNPs. Additionally, functional genomics approaches, such as transcriptomic or proteomic analyses in contrasting genotypes, would provide key insights into the molecular pathways associated with these loci, helping to determine the biological relevance of candidate genes and to translate this information into effective strategies for breeding and improvement.

Finally, it is important to note that while selection is a plausible driver of elevated $F_{\mathrm{ST}}$ at certain loci, demographic processes such as drift, bottlenecks, and historic gene flow can also produce similar signatures. A better understanding of these processes will require integration of demographic modeling analyses in future studies.

### 4.4 Final remarks and perspectives

Our integrative approach provides a high‐resolution perspective of the forces shaping phenotypic and genetic diversity in cultivated alfalfa. While phenotypic structure is detectable, it does not fully align with genetic differentiation, highlighting the need for combined analyses to guide breeding strategies more effectively. Future improvements in yield, persistence, and stress tolerance will depend not only on deciphering trait architecture through classical approaches such as GWAS and genomic selection, but also on identifying adaptive alleles across divergent gene pools, as found in this study.

While this work advances our understanding of genotype–phenotype relationships in alfalfa, it also highlights the need for robust follow‐up studies. Functional genomics, including gene expression analyses and genetic validation using segregating populations or panels with contrasting allele frequencies for outlier SNPs, will be essential for confirming the relevance and breeding value of these loci. Integrating these approaches will be critical for translating candidate gene discoveries into effective tools for marker‐assisted and genomic selection.

Overall, this work clarifies long‐standing questions about the relationship between genetic structure and phenotypic diversity in alfalfa, and highlights specific trait–gene associations of direct value for breeding. These insights provide a foundation for the development of cultivars with greater resilience, productivity, and adaptability, contributing to more sustainable forage production worldwide.

## AUTHOR CONTRIBUTIONS

**Irving Arcia‐Ruiz**: Data curation; formal analysis; investigation; methodology; resources; software; validation; visualization; writing—original draft. **Marie Pégard**: Data curation; formal analysis; investigation; methodology; resources; software; supervision; validation; writing—review and editing. **Fabien Surault**: Investigation; methodology; resources; writing—review and editing. **Dalibor Živanov**: Data curation; investigation; methodology; writing—review and editing. **Dragan Milić**: Investigation; methodology; resources; writing—review and editing. **Đura Karagić**: Conceptualization; funding acquisition; methodology; project administration; resources; writing—review and editing. **Bernadette Julier**: Conceptualization; formal analysis; funding acquisition; investigation; methodology; project administration; resources; supervision; validation; writing—review and editing.

## CONFLICT OF INTEREST STATEMENT

The authors declare no conflicts of interest.

## Supporting information



Table S1. Composition and geographic origin of the *Medicago sativa* L. diversity panel.

Table S2. List of phenotypic traits, measurement dates and trial locations.

Table S3. Importance scores of all phenotypic traits for genetic group differentiation according to Boruta feature selection.

Table S4. Highly differentiated SNPs (F_ST_ outliers) and gene annotations identified across pairwise genetic group comparisons in cultivated alfalfa.

Supplemental figures are provided in File S1, and supplemental tables are provided as separate files (Table S1–S4), all of which are included with this manuscript.

## Data Availability

The genotyping data are available at https://doi.org/10.57745/L0FLJD. The phenotyping data are available at https://doi.org/10.57745/S1YDX1.
