# Supplementary material for: Phenotypic and genomic insights into alfalfa diversity: Identifying critical loci for enhanced resilience
Source: Plant Genome. 2025 Nov 19;18(4):e70155. doi: 10.1002/tpg2.70155 (PMC12631055; doi:10.1002/tpg2.70155)
Supplement: Supplementary file 5 — Supplemental figures are provided in File S1, and supplemental tables are provided as separate files (Table S1–S4), all of which are included with this manuscript. [file TPG2-18-e70155-s003.docx]

**File S1 with supplementary Figures A, B, C, D, and E.**


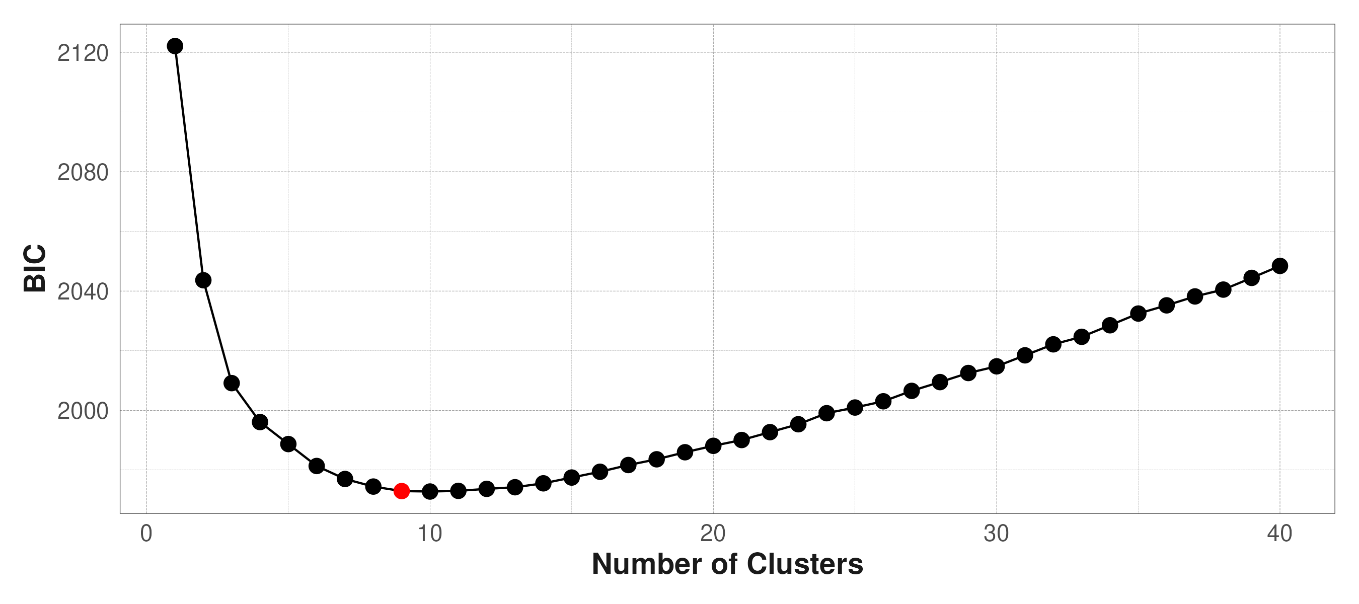


**Figure A.** **Bayesian Information Criterion (BIC) curve for determining the optimal number of phenotypic groups.** The BIC values were calculated for different numbers of groups (k) from 1 to 40. The optimal number of groups (k=9) is colored in red and corresponds to the lowest BIC value, indicating the best model fit for the data. The curve illustrates the stability of the clustering solution with respect to the number of groups.


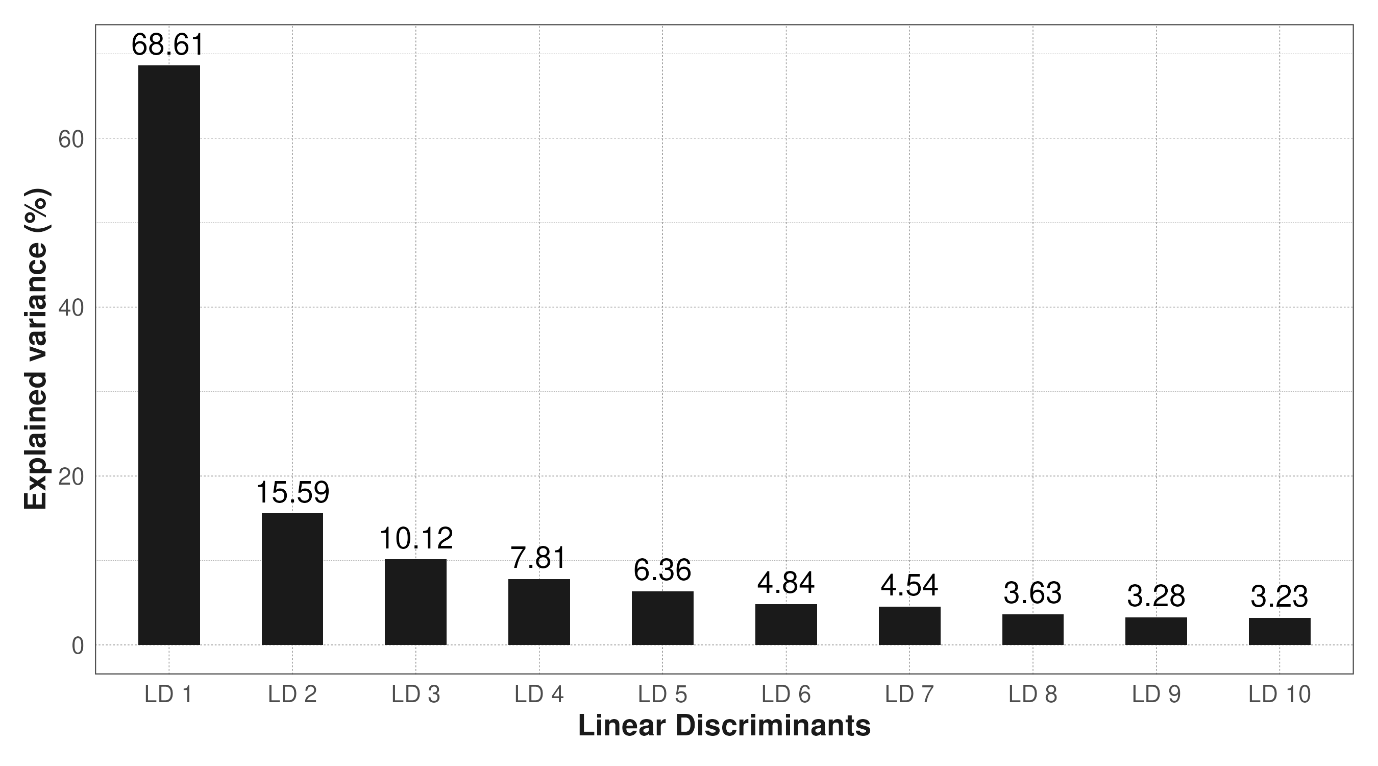


**Figure B. Variance explained by the first ten discriminant axes from Discriminant Analysis of Principal Components (DAPC).** Each bar represents the percentage of variance explained by one of the first ten discriminant axes from the DAPC performed on the phenotypic dataset.


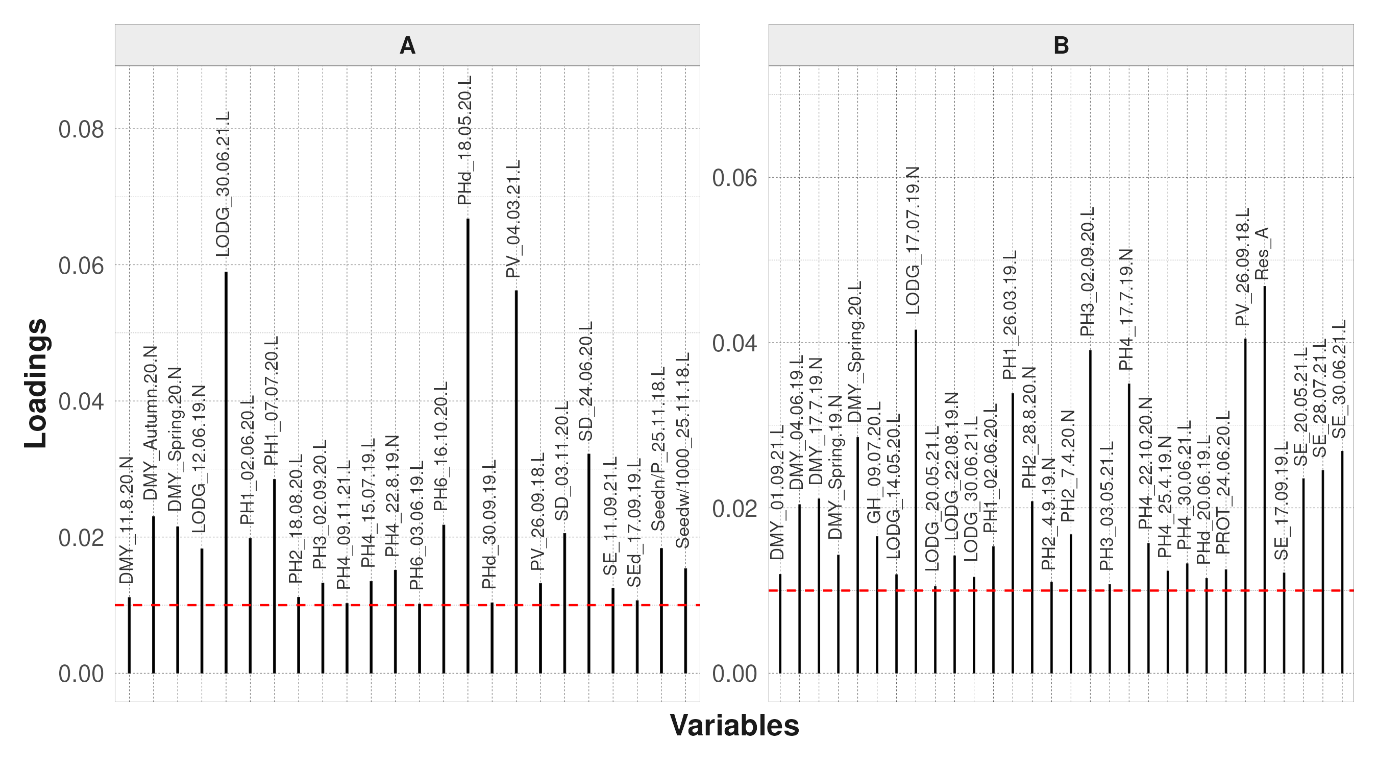


**Figure C. Phenotypic traits contributing to differentiation along the axes of the principal component analysis.** (A) First axis. (B) Second axis. Only variables with loadings above the 0.10 threshold are shown. A dotted line indicates the threshold used for selection.


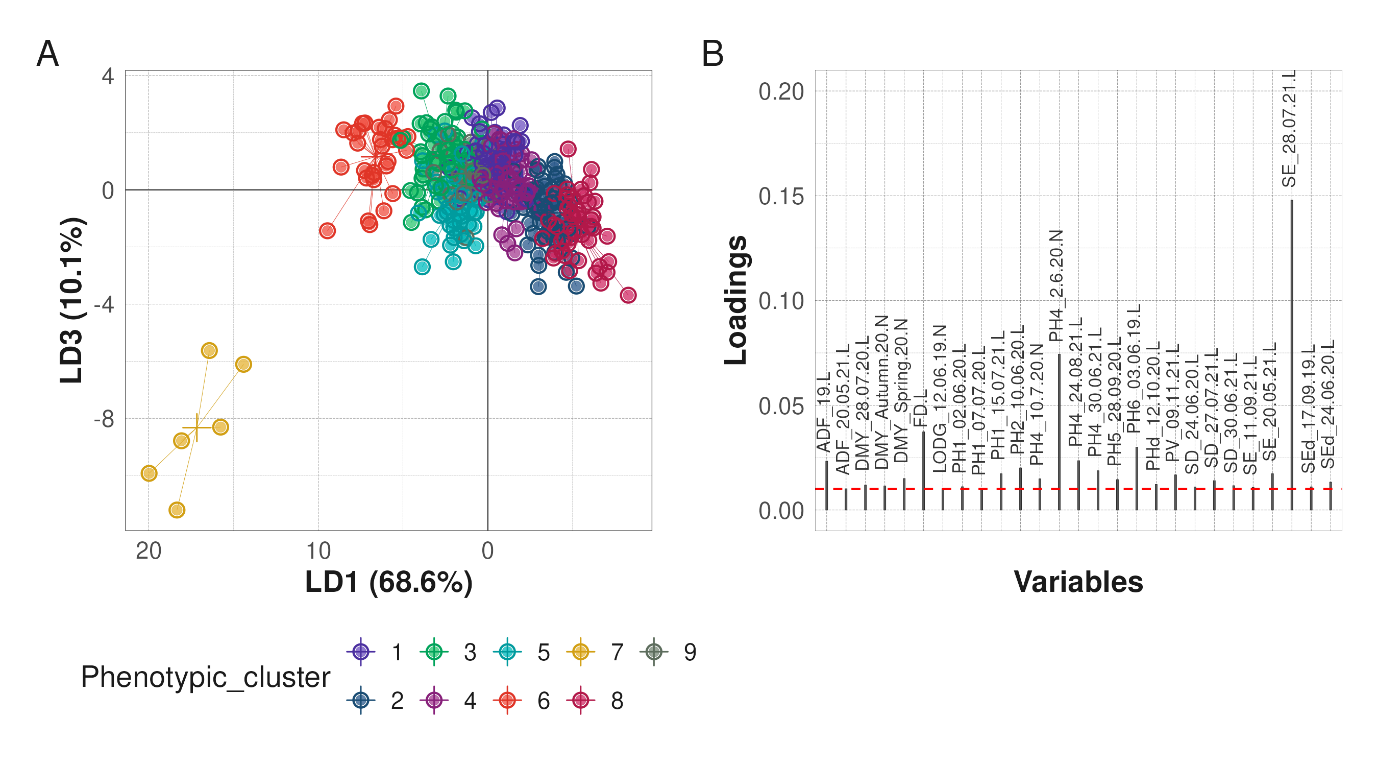


**Figure D. Discriminant analysis of phenotypic data based on the first and third axes.** (A) Projection of accessions onto the first and third discriminant axes, showing the distribution of accessions across these axes. Accessions are color-coded according to their phenotypic groups identified by k-means clustering. (B) Key phenotypic traits contributing to differentiation along the third axe. Only variables with loadings above the 0.10 threshold are shown, with a dotted line indicating the threshold used for selection.


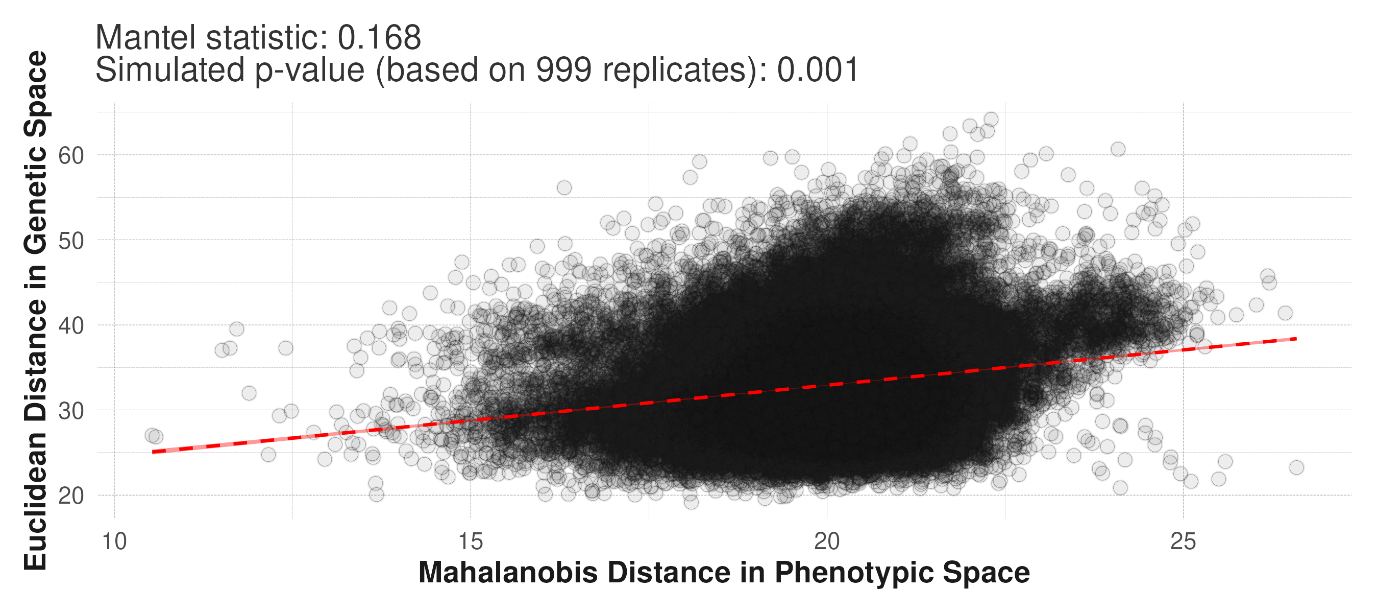


**Figure E. Relationship between genetic and phenotypic distances.** Scatter plot showing the relationship between pairwise genetic distances (Euclidean metric) and phenotypic distances (Mahalanobis metric) for unique accession pairs. Each point represents a unique pairwise comparison between accessions. The red dashed line indicates the linear regression fit.
